# The C825T Polymorphism of the G-Protein β3 Gene as a Risk Factor for Depression: A Meta-Analysis

**DOI:** 10.1371/journal.pone.0132274

**Published:** 2015-07-06

**Authors:** Liang Fang, Chanjuan Zhou, Shunjie Bai, Chenglong Huang, Junxi Pan, Ling Wang, Xinfa Wang, Qiang Mao, Lu Sun, Peng Xie

**Affiliations:** 1 Department of Neurology, Yongchuan Hospital, Chongqing Medical University, Chongqing, China; 2 Department of Neurology, the First Affiliated Hospital of Chongqing Medical University, Chongqing, China; 3 Chongqing Key Laboratory of Neurobiology, Chongqing, China; 4 Institute of Neuroscience, Chongqing Medical University, Chongqing, China; 5 Key Laboratory of Laboratory Medical Diagnostics, Ministry of Education, Department of Laboratory Medicine, Chongqing Medical University, Chongqing, China; Peking University, CHINA

## Abstract

**Background:**

TheG-protein β3 gene (GNβ3) has been implicated in psychiatric illness through its effects upon intracellular transduction of several neurotransmitter receptors. Multiple studies have investigated the relationship of the C825T polymorphism of the GNβ3 gene (GNβ3 C825T) to depression and antidepressant response. However, the relationship between GNβ3 C825T and depression remains inconsistent. Therefore, here we performed a meta-analysis to investigate the role of GNβ3 C825Tin depression risk.

**Methods:**

Published case-control studies examining the association between GNβ3 C825T and depression were systematically searched for through several electronic databases (PubMed, Scopus, Science Direct, Springer, Embase, psyINFO, and CNKI). The association between GNβ3 C825T and depression risk were assessed by odd ratios (ORs) and their 95% confidence intervals (CIs) for each study. Pooled ORs were constructed for allele contrast (C versus T), homozygote (CC versus TT) model, heterozygote (CC versus CT) model, dominant model (CC + CT versus TT), and recessive (CC versus TT+CT) model. In order to evaluate possible biases, a sensitivity analysis was conducted by sequential deletion of individual studies in an attempt to assess the contribution of each individual dataset to the pooled OR.

**Results:**

Nine studies, including 1055 depressed patients and 1325 healthy controls, were included. A significant association between GNβ3 C825Tand depression was found to exist, suggesting that the T-allele of GNβ3 C825Tcan increase susceptibility to depression. After stratification by ethnicity, the same association was found in the Asian subpopulation, but not the Caucasian subpopulation.

**Conclusions:**

This is the first meta-analysis to reveal a relationship between GNβ3 C825T and depression. Asian T-allele carriers of GNβ3 C825T appear to be more susceptible to depression.

## Introduction

Major depressive disorder (MDD) is a prevalent psychiatric disorder characterized by persistent depressed mood and anhedonia [1]. According to clinical and animal model research, different aspects of human physiology are altered in depression, including the neurotransmitter and neuropeptide systems, neurotropic factors, the hypothalamic-pituitary-adrenal (HPA) axis, and hippocampal neurogenesis [2–4].

In particular, reduced G-protein function has been identified in the peripheral blood cells of patients with depression [5], and altered levels of G-proteins have been found in two regions of the prefrontal cortex of depressed human subjects, which were attenuated by antidepressant therapy [6,7]. G-proteins play key roles in molecular signaling following neurotransmitter receptor activation, leading to an increase of intracellular calcium ion (Ca^2+^) concentrations [8–10]. On this basis of modulating neurotransmitter receptor activation, G-proteins may be one of the keys to understanding the underlying mechanism(s) of depression [11].

In recent years, several genome-wide association studies (GWAS) have discovered statistically significant genetic variations relevant to the etiology of depression, yielding novel insights into genetic risk factors underlying depression [12,13]. Several genetic polymorphisms, such as BDNF Val66Met and 5-HTR2A T102C, have been identified as potential risk factors for depression [14,15]. Another such polymorphism—the C825T polymorphism within the G-protein β3 gene (GNβ3 C825T)—has been increasingly linked to depression. The T-allele of GNβ3 C825T can result in the deletion of 41 amino acids, leading to alterations in cellular signal transduction and ion transport [16]. The association between GNβ3 C825T and depression was first identified through a polymerase chain reaction (PCR)-based method [17], which has been followed by additional GNβ3 genotyping studies across different countries worldwide.

However, results from these genotyping studies have been contradictory. While some studies have found that the frequency of the T-allele of GNβ3 C825T is significantly higher in depressed patients, several other studies have shown no associations between depression and GNβ3 gene polymorphisms. Therefore, here we performed a meta-analysis to assess the relationship between depression and GNβ3 C825T.

## Methods

### Search Strategy and Inclusion Criteria

All published studies examining the association between GNβ3 C825T and depression were systematically searched for through several electronic databases (PubMed, Scopus, Science Direct, Springer,Embase, psyINFO, and CNKI) from January 1990 to September 2014 using the following search terms: (“G protein-β-3” OR GNβ3) AND C825T AND (“mood disorders” OR “major depressive disorder” OR MDD OR “depressive episode” OR “depression”).

Only full-length articles meeting the following criteria were included: (i) a case-control design; (ii) evaluating GNβ3 C825T and depression risk; (iii) an adequate description of the diagnostic criteria for patient inclusion and exclusion; and (iv) sufficient reported data for estimating odds ratios (ORs) and their 95% confidence intervals (95% CIs). Abstracts, conference proceedings, case studies, family-based designs, population-based studies of healthy subjects, reviews, and duplicate cohorts were excluded.

### Data Extraction

Three authors independently extracted data to avoid extraction errors with discrepancies resolved by discussion. The following parameters were extracted from each eligible article: first author, publication year, country of origin, ethnicity (defined as either Asian or Caucasian), diagnostic system, number of cases and controls (male/female), antidepressant therapy, Hardy-Weinberg equilibrium, the available genotype, and allele frequency information for the C825T polymorphism.

### Statistical Methods

All statistical analyses were conducted using Rev Man 5.0.1 and STATA software (version 12.1; Stata Corporation, College Station, Texas, USA). All *P*-values were two-sided with a *P*<0.05 considered statistically significant. The association between GNβ3 C825T and depression risk were assessed by ORs (and their 95% CIs) for each study. Pooled ORs were constructed for allele contrast (C versus T), homozygote (CC versus TT) model, heterozygote (CC versus CT) model, dominant model (CC + CT versus TT), and recessive (CC versus CT+TT) model. A chi-squared-based Q-statistic test was used to detect the heterogeneity among studies. If the *P*-value of the Q-test exceeded 0.05 (indicating a lack of heterogeneity among the studies), a fixed-effect model was used; otherwise, a random-effects model was used. We used a *Z*-test to determine the significance of the pooled ORs with a *P*<0.05 considered statistically significant.

In order to evaluate possible biases, a sensitivity analysis was conducted by sequential deletion of individual studies in an attempt to assess the contribution of each individual dataset to the pooled OR. Finally, we estimated publication bias by Egger’s test with a *P*<0.05 considered statistically significant.

## Results

### Literature Search Results

**Fig 1:**
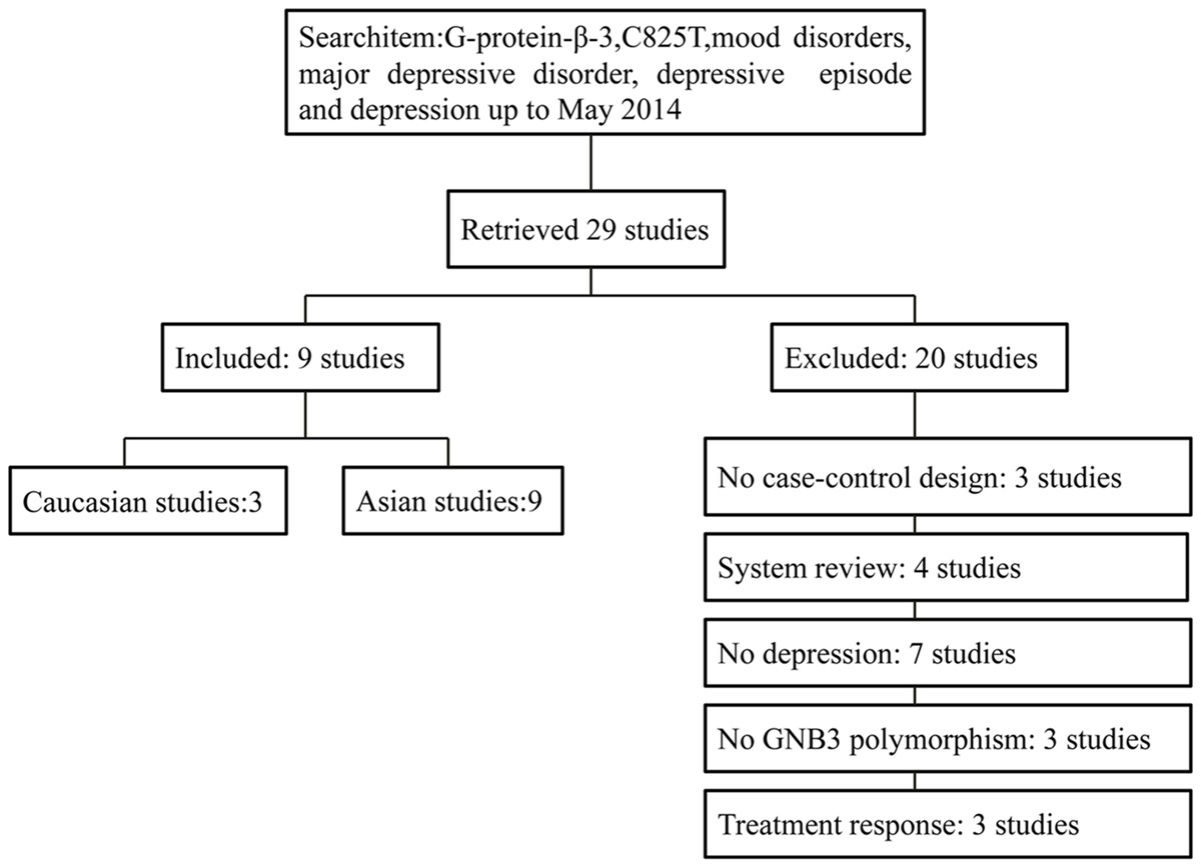
Flowchart of Study Selection.

The study selection procedure is shown in Fig 1. The literature search identified 230 potentially relevant records. After screening titles and abstracts, 29 full-text articles were reviewed, of which 20 were excluded for the following reasons: (i) four studies were systematic reviews or meta-analyses on G-protein function [6,11,18,19]; (ii) seven studies assessed SNP effects in other psychiatric disorders [20–26]; (iii) three studies did not use a case-control design [27–29]; (iv) three studies did not assess GNβ3 C825T but measured G-protein expression [5,30,31]; and (v) three studies assessed GNβ3 C825T and the antidepressant response [16,32,33]. There were no previously published GWAS concerning GNβ3 C825T in depression, so no GWAS was included in this meta-analysis.

Hence, nine studies were ultimately included in this meta-analysis based on our inclusion criteria [34–42]. The study characteristics are displayed in Tables 1 and 2. Of these nine included studies, three were on Caucasians and the other six were on Asians. The genotype distributions were in agreement with the Hardy-Weinberg equilibrium for each individual study.

**Table 1:** Key Characteristics of Included Studies.

Author	Year	Country	Ethnicity	Diagnosis	Ratingscale	Controls(F)mean age±SD	Depression(F)mean age±SD	Genotyping method
Alessandro	2012	Italy	Caucasian	MDD	-	76-	222(161)50.06±15.02	-
Anttila	2007	Finland	Caucasian	Depression	-	392(182)44.4±11.1	119(65)57.7±14.0	Taq ManAssay
Cao	2007	China	East Asian	Depression	HAMD-17≥18	156(80)54.44±6.542	180(96)55.84±8.522	PCR-RFLP
Chen	2011	China	East Asian	PSD	HAMD-24≥21	106(34)60.7 ±13.2	53(20)62.9 ±13.8	PCR-RFLP
Kunugi	2002	Japan	East Asian	Depression	-	198(104)30.0 ± 8.1	68(44)54.6 ± 14.1	PCR-RFLP
Lee	2004	Korea	East Asian	MDD	HAMD-17>18	133(89)43.4±10.2	106(78)47.1±13.3	PCR-RFLP
Lin	2001	Taiwan	East Asian	Depression	-	153(72)39.8 ±18.1	65(40)39.8 ± 13.7	PCR-RFLP
Peter	2000	Germany	Caucasian	Depression	HAMD-1726.7 ±6.4	111(57)47.3 ±12.1	88(59)51.6 ±13.0	PCR-RFLP
Xiao	2002	China	East Asian	Depression	HAMD-17>17	100(50)28±7	154(93)43±14	PCR-RFLP

MDD: major depressive disorder; HAMD: Hamilton Depression Rating Scale; PSD: post-stroke depression.

**Table 2:** Genotyping Characteristics of Included Studies.

Author	Diagnosis	Cases	Genotype distribution (%)			Allele frequency (%)		HWE
			CC	CT	TT	C	T	
Alessandro	Control	76	36(47)	31(41)	9(12)	103(68.0)	49(32.0)	Yes
	MDD	222	86(39)	115(52)	21(9)	287(65.0)	157(35.0)	
Anttila	Control	392	218 (55.6)	144 (36.7)	30 (7.7)	580(74.0)	204(26.0)	Yes
	Depression	119	63 (52.9)	46 (38.7)	10 (8.4)	172(72.3)	66(27.7)	
Cao	Control	156	44(28.2)	72(46.2)	40(25.6)	160(51.3)	152(48.7)	Yes
	Depression	180	20(11.1)	76(42.2)	84(46.7)	116(32.2)	244(67.8)	
Chen	Controls	106	29 (27.4)	41 (38.7)	36(34.0)	99(46.7)	113 (53.3)	Yes
	PSD	53	8(15.1)	22 (41.5)	23 (43.4)	38 (35.8)	68 (64.2)	
Kunugi	Control	198	49 (24.7)	90 (45.5)	59 (29.8)	188 (47.5)	208 (52.5)	Yes
	Depression	68	16 (23.5)	32 (47.1)	20 (29.4)	64 (47.1)	72 (52.9)	
Lee	Control	133	43 (32.3)	62 (46.6)	28 (21.1)	148(56.0)	118(44.0)	Yes
	MDD	106	19 (17.9)	60 (56.6)	27 (25.5)	98(46.0)	114(54.0)	
Lin	Control	153	31 (20.0)	90 (59.0)	32 (21.0)	152(52.0)	154(48.0)	Yes
	Depression	65	16 (25.0)	36 (55.0)	13 (20.0)	68(52.3)	62(47.7)	
Peter	Control	111	57 (52.0)	46 (41.0)	8 (7.0)	160(72.0)	62(28.0)	Yes
	Depression	88	33 (38.0)	36 (41.0)	19 (21.0)	102(58.0)	74(42.0)	
Xiao	Control	100	27(27.0)	51(51.0)	22(22.0)	105(52.5)	95(47.5)	Yes
	Depression	154	35(22.7)	49(31.8)	70(44.8)	119(38.6)	189(61.4)	

HWE: Hardy-Weinberg equilibrium; MDD: major depressive disorder; PSD:post-stroke depression.

### Overall Meta-Analysis

**Fig 2:**
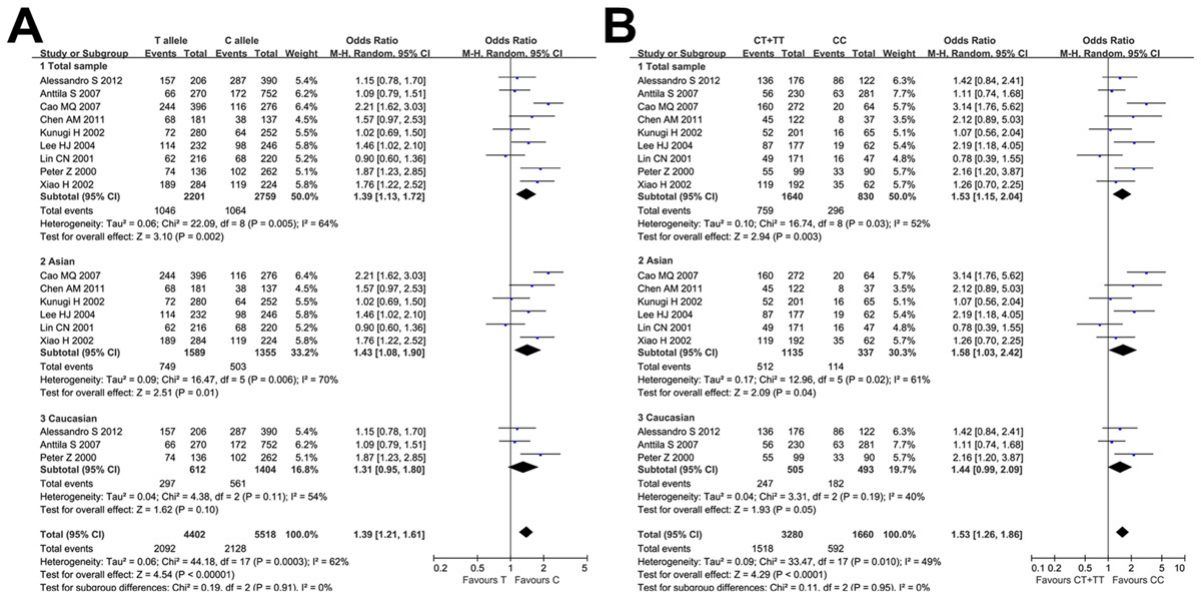
Meta-Analyses for the Association between the GNβ3 C825T Polymorphism and Depression. Overall and subgroup forest plots showing the summary effect sizes and heterogeneity findings for (A) C-allele versus T-allele and (B) the recessive model (CC versus CT+TT).

**Fig 3:**
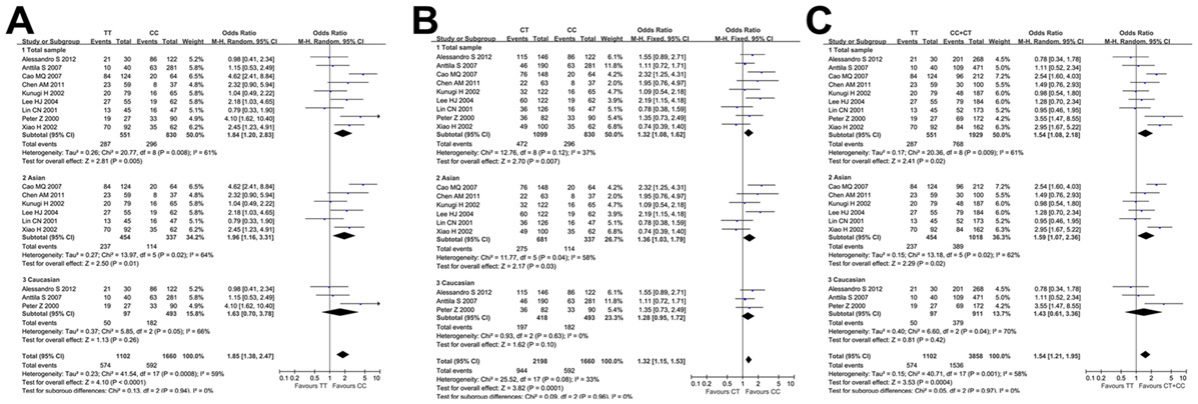
Further Meta-Analyses for the Association between the GNβ3 C825T Polymorphism and Depression. Overall and subgroup forest plots showing the summary effect sizes and heterogeneity findings for (A) TT homozygosity versus CC homozygosity, (B) the heterozygote model (CC versus CT), and (C) the dominant model (CC + CT versus TT).

The nine case-control studies, consisting of 1055 depressed cases and 1325 controls, were pooled together to assess the association between depression and GNβ3 C825T. On the basis of the random effects model, the pooled OR for the T-allele of GNβ3 C825T showed a significant correlation with depression risk under the allele model (C-allele versus T-allele: OR = 1.39, 95% CI = 1.13–1.72, Z = 3.10, *P* = 0.002; Fig 2). When we calculated the pooled OR for TT homozygosity relative to CC homozygosity, the OR increased to 1.84 (95% CI = 1.20–2.83, Z = 2.81, *P* = 0.005; Fig 3). Significant associations between the T-allele of GNβ3 C825T and depression risk were also observed under the dominant model (CC + CT versus TT: OR = 1.54, 95% CI = 1.08–2.18, *P* = 0.02), the recessive model (CC versus CT+TT: OR = 1.53, 95% CI = 1.15–2.04, *P* = 0.02), and the heterozygote model (CC versus CT: OR = 1.32, 95% CI = 1.08–1.62, *P* = 0.03; Figs 2 and 3).

### Subgroup Analysis

A subgroup analysis was performed based on ethnicity. The ethnicity-stratified analysis indicated that GNβ3 C825T is strongly related to depression risk in the Asian subpopulation under all genetic models except for the heterozygote model (CC versus CT: OR = 1.35, 95% CI = 0.87–2.08, *P* = 0.18; Table 3). However, no relationship between GNβ3 C825T and depression was found in Caucasian subpopulation under any genetic model (Figs 2 and 3).

**Table 3:** Odds Ratios and 95% Confidence Intervals for the Association between the GNβ3 C825T Polymorphism and Depression.

	C-allele vs. T-allele		CC vs. TT		CC vs. CT		CC vs. CT + TT		CC + CT vs. TT	
	OR (95% CI)	*P*	OR (95% CI)	*P*	OR (95% CI)	*P*	OR (95% CI)	*P*	OR (95% CI)	*P*
Overall	1.39(1.13, 1.72)	0.002	1.84 (1.20, 2.83)	0.005	1.32(1.08, 1.62)	0.007	1.53(1.15,2.04)	0.003	1.54 (1.08, 2.18)	0.02
By ethnicity										
Caucasian	1.31 (0.95, 1.80)	0.10	1.63(0.70, 3.78)	0.26	1.28(0.95, 1.72)	0.11	1.44(0.99, 2.09)	0.051	1.43 (0.61, 3.36)	0.42
East Asian	1.43 (1.08, 1.90)	0.01	1.96(1.16, 3.31)	0.01	1.35(0.87, 2.08)	0.18	1.58 (1.03, 2.42)	0.04	1.59(1.07, 2.36)	0.02

### Heterogeneity Analysis

Significant heterogeneity was found among ORs in overall comparisons (I^2^ = 64%, Tau^2^ = 0.06 for allele model; I^2^ = 61%, Tau^2^ = 0.26 for homozygote model; I^2^ = 61%, Tau^2^ = 0.17 for dominant model), while no heterogeneity was found under the heterozygote model (I^2^ = 37%, Tau^2^ = 12.76). To determine the origins of the heterogeneity, subgroup analysis on ethnicity was carried out as described above. However, significant heterogeneity remained among the Asian and Caucasian subpopulations.

### Sensitivity and Publication Bias Analysis

Sensitivity analyses were conducted with the leave-one-out method to assess the degree to which each individual study influenced the results of the overall analysis. The results of the sensitivity analysis confirmed that no single study influenced the pooled ORs (S1–S5 Tables). No strong statistical evidence for publication bias was observed in Egger׳s test (all *P*>0.05) (S1–S5 Figs).

## Discussion

To our knowledge, this is the first meta-analysis to demonstrate a relationship between GNβ3 C825T and depression. We used 5 models to estimate the relationship between G protein-β-3 gene C825T polymorphism and depression. A significant association between T-allele withinGNβ3 C825T and depression were found both in the homozygote and heterozygote genotype variation. The results of the dominant model and the recessive model supported CT genotype and TT genotype respectively could increase the risk of depression. Notably, compared with cohorts without the variation, the frequency of the GNβ3 C825T TT genotypes in depressed patients was significantly higher than that of healthy controls with an increase of depression by 84 percent; the heterozygote variation (CT) caused an increase of depression by 32 percent as well. The results of our meta-analysis among all the 5 models showed that GNβ3 C825T polymorphism increased a risk of depression and the sensitivity analyses further confirmed the stability of the results, suggesting that GNβ3 C825T may be an important heritable factor underlying the genetic mechanism of depression. Our results also show a significant association between the T-allele of GNβ3 C825T and depression risk in Asians, but not in Caucasians.

GNβ3 C825T has been shown to be predictive of depressive mood in a young, healthy Western population [29], and previous German studies [17,29,41] report that T-allele carriers of GNβ3 C825T are more prone to depression. In contrast to these previous studies, our results show that the C825T polymorphism does not show any relationship with depression risk in Caucasians. In accordance with our findings, a previous meta-analysis performed by Hu et al.[19] found that GNβ3 C825T has no effect on the antidepressant response to MDD in Caucasians. Rosskopf et al.’s analysis of GNβ3 gene polymorphisms in Caucasians, Africans, and Asians [43] found that the prevalence of GNβ3 haplotypes in these various ethnic populations differs. Notably, the two key GNβ3 polymorphisms, termed 'C-haplotype' and 'T-haplotype', were restricted to one or two major ethnic populations. As higher T-allele frequencies of GNβ3 C825T are found in Asians over Caucasians, we speculate that ethnogenetic heterogeneity in T-allele frequencies may underlie these observed discrepancies between Asians and Caucasians.

Thus far, the majority of psychiatric studies have focused on investigating the function and expression of G-proteins in affective disorders. G-proteins are composed of three subunits, which can dissociate into G_α_ and G_βγ_ units after receptor activation. The G_β_ subunit is further subdivided into three subtypes: 1, 2, and 3 [44,45]. Significant elevations in the stimulatory G_α_ subunit (G_αs_) have been observed in peripheral blood cells and post-mortem brain tissue from bipolar depressed patients [46]. Moreover, peripheral blood cells demonstrate elevated platelet levels of G_α_ in patients with unipolar major depression [30].

Ever since Siffert et al. first identified a genetic variant (C825T) in exon 10 of the G-protein gene [47], GNβ3 C825T has become one of the most investigated genetic variations in bipolar depression and major depression [20,37]. Previous studies have attempted to determine the association between GNβ3 C825T and antidepressant response in MDD patients. Since disparate conclusions exist from these studies, Hu et al. performed a meta-analysis, including seven studies composed of 1047 depressed patients, to assess this question [19]. His research group showed that GNβ3 C825T may influence antidepressant response to MDD among Asians. Accordingly, our meta-analysis demonstrates that GNβ3 C825T may be a possible risk factor for depression in Asians. As GNβ3 C825T has been previously associated with monoamine neurotransmitter receptor activation [48], the altered signal transduction produced by the T-allele of GNβ3 C825 may underlie the findings from Hu et al.’s and our meta-analyses. These findings may provide genetic target(s) to explore the underlying mechanism of depression and aid in the development of more effective antidepressants.

Significant heterogeneity was found among ORs in the allele model, homozygote model, and dominant model. Possible factors underlying this high heterogeneity may include age, gender, and ethnicity. However, no differences were detected after an ethnicity-based subgroup analysis. Gender differences were also considered; however, due to the lack of reported data, we could not perform this analysis. Notably, Anttila et al. has previously identified an association between GNβ3 C825T and depression risk in females but an opposing trend in males [34]. Clearly, larger clinincal studies on GNβ3 C825T and depression risk with age-based, gender-based, and ethnicity-based subgroups are necessary to analyze these factors.

Several limitations should be mentioned with respect to our findings. Firstly, the number of included studies was not sufficient for a comprehensive analysis of GNβ3 C825T and depression risk in the Caucasian subpopulation. Thus, more studies are needed to explore the relationship between GNβ3 C825T and depression in Caucasians. Secondly, only English studies were included in the meta-analysis. This may have been a source of publication bias although no such publication bias was found in our meta-analysis. Thirdly, we did not analyze the possible impact of gender differences, which may explain the observed heterogeneity. Finally, one study by Chen et al., which mainly targeted PSD patients, was not excluded from this study, as it could be classified into depression. The sensitivity analyses indicated that this study did not influence the effect size or conclusions.

In conclusion, this is the first meta-analysis to reveal a relationship between GNβ3 C825T and depression. We found that Asian T-allele carriers of GNβ3 C825T are more susceptible to depression. In contrast, no significant association between T-allele carriers of GNβ3 C825T and depression risk was found in Caucasians. These results may provide clinicians and public health administrators with an important screening tool for assessing depression. As many factors have been associated with depression risk, additional factors (such as gender, age, ethnicity, and environmental factors) should be taken into consideration in future studies on this topic.

## Supporting Information

S1 Fig Egger’s Test for C-Allele versus T-Allele. (TIF)

S2 Fig Egger’s Test for CC versus CT+TT. (TIF)

S3 Fig Egger’s Test for CC versus TT. (TIF)

S4 Fig Egger’s Test for CC versus CT. (TIF)

S5 Fig Egger’s Test for CC+CT versus TT. (TIF)

S1 File PRISMA 2009 Flow Diagram. (DOC)

S2 File PRISMA 2009 Checklist. (DOC)

S3 File Meta-analysis-on-genetic-association-studies-form. (DOCX)

S1 Table Sensitivity Analyses for C-Allele versus T-Allele. (DOCX)

S2 Table Sensitivity Analyses for CC vs. CT+TT. (DOCX)

S3 Table Sensitivity Analyses for CC vs. TT. (DOCX)

S4 Table Sensitivity Analyses for CC vs. CT. (DOCX)

S5 Table Sensitivity Analyses for CC+CT vs. TT. (DOCX)
