# Supplementary figures and images for: The C825T Polymorphism of the G-Protein β3 Gene as a Risk Factor for Depression: A Meta-Analysis
Source: PLoS One. 2015 Jul 6;10(7):e0132274. doi: 10.1371/journal.pone.0132274 (PMC4493085; doi:10.1371/journal.pone.0132274)

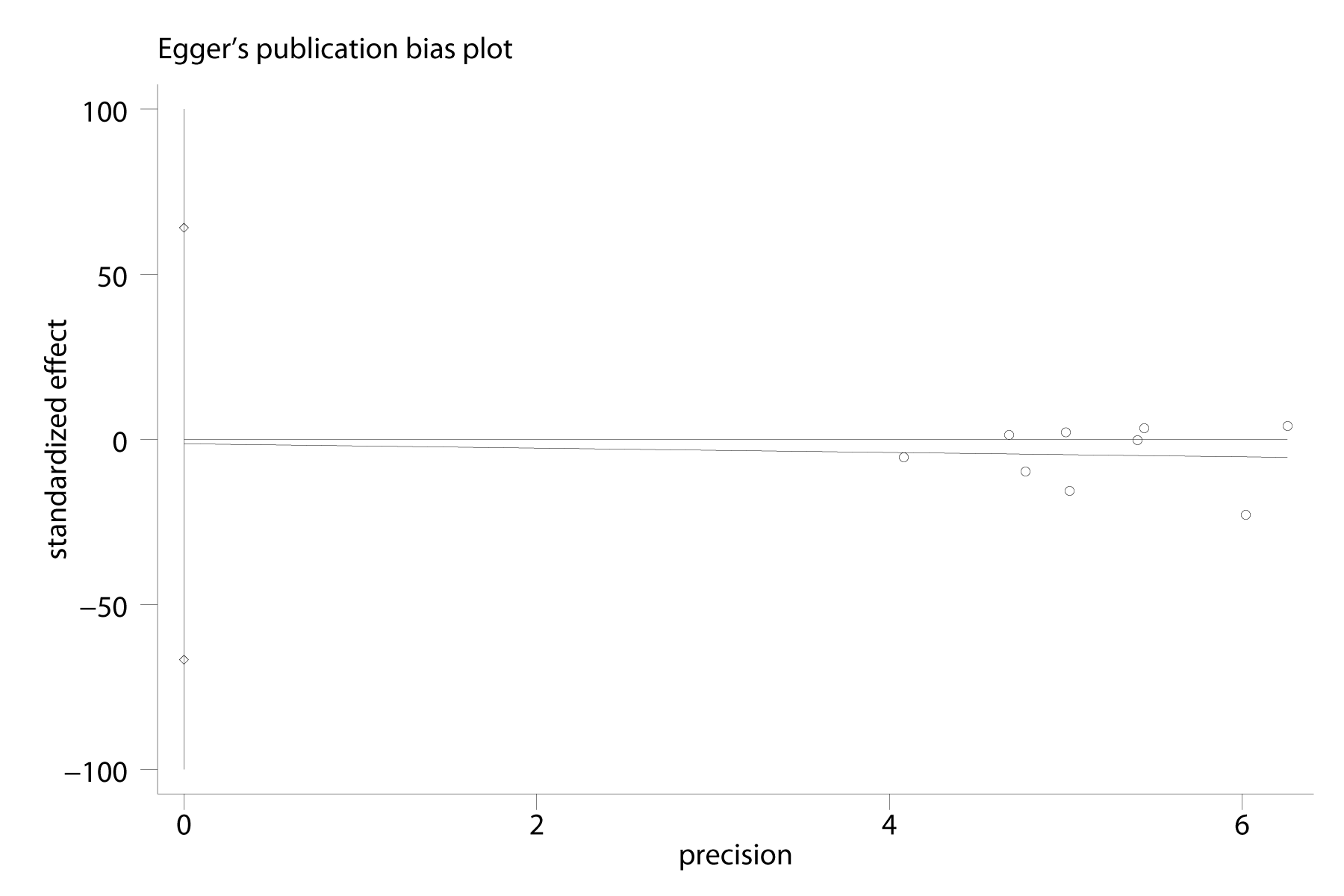

Supplement: S1 Fig — (TIF) [file pone.0132274.s001.tif]

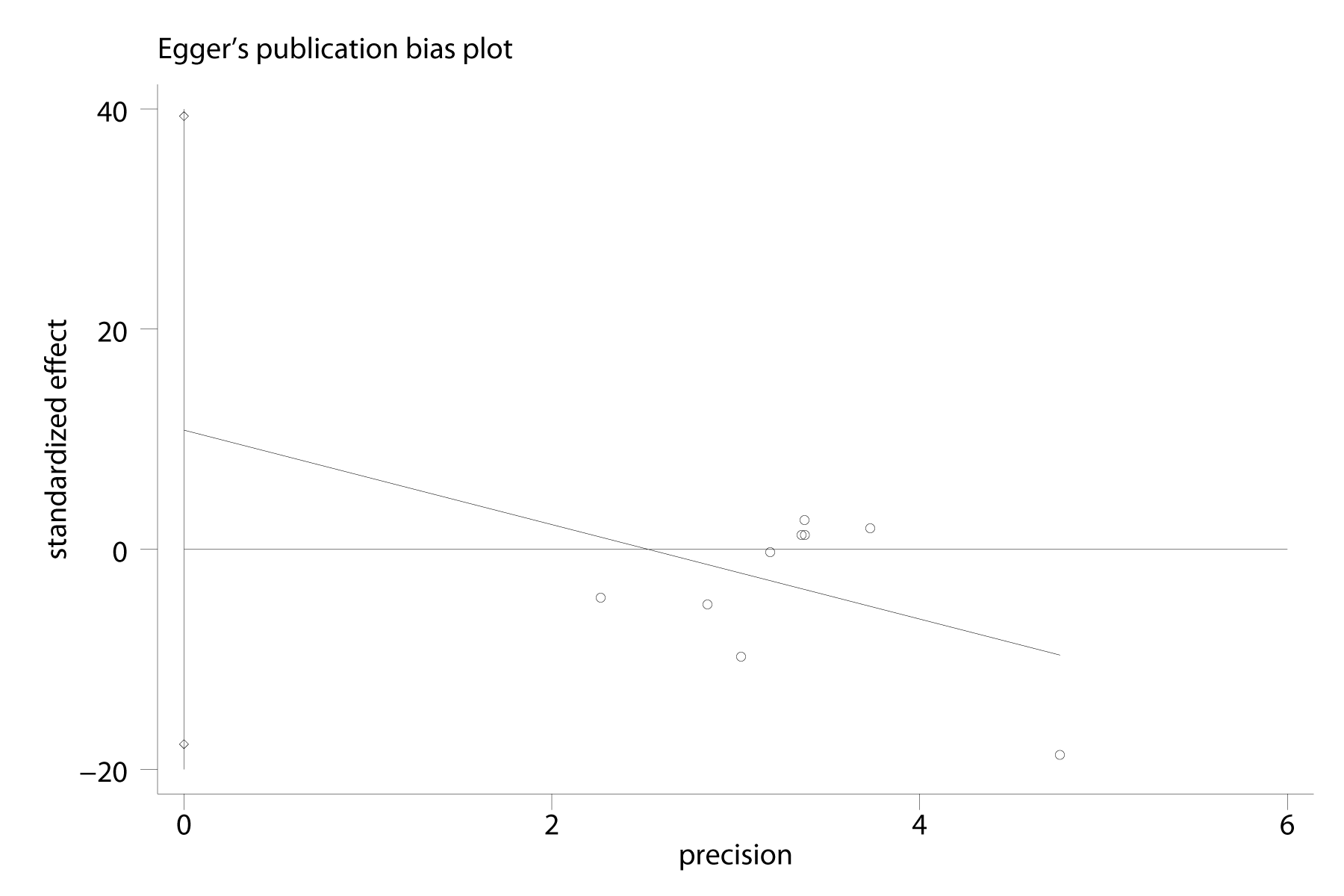

Supplement: S2 Fig — (TIF) [file pone.0132274.s002.tif]

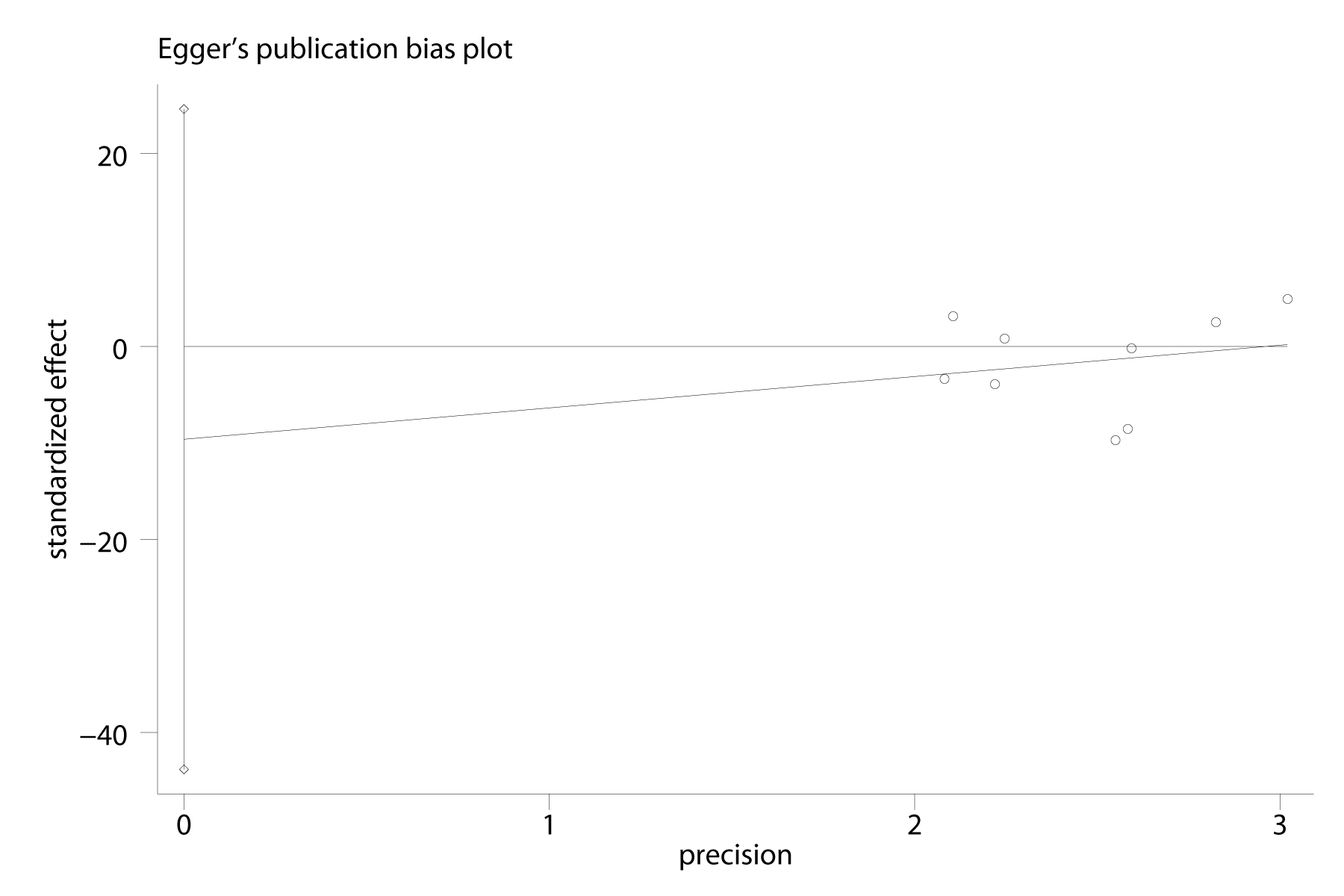

Supplement: S3 Fig — (TIF) [file pone.0132274.s003.tif]

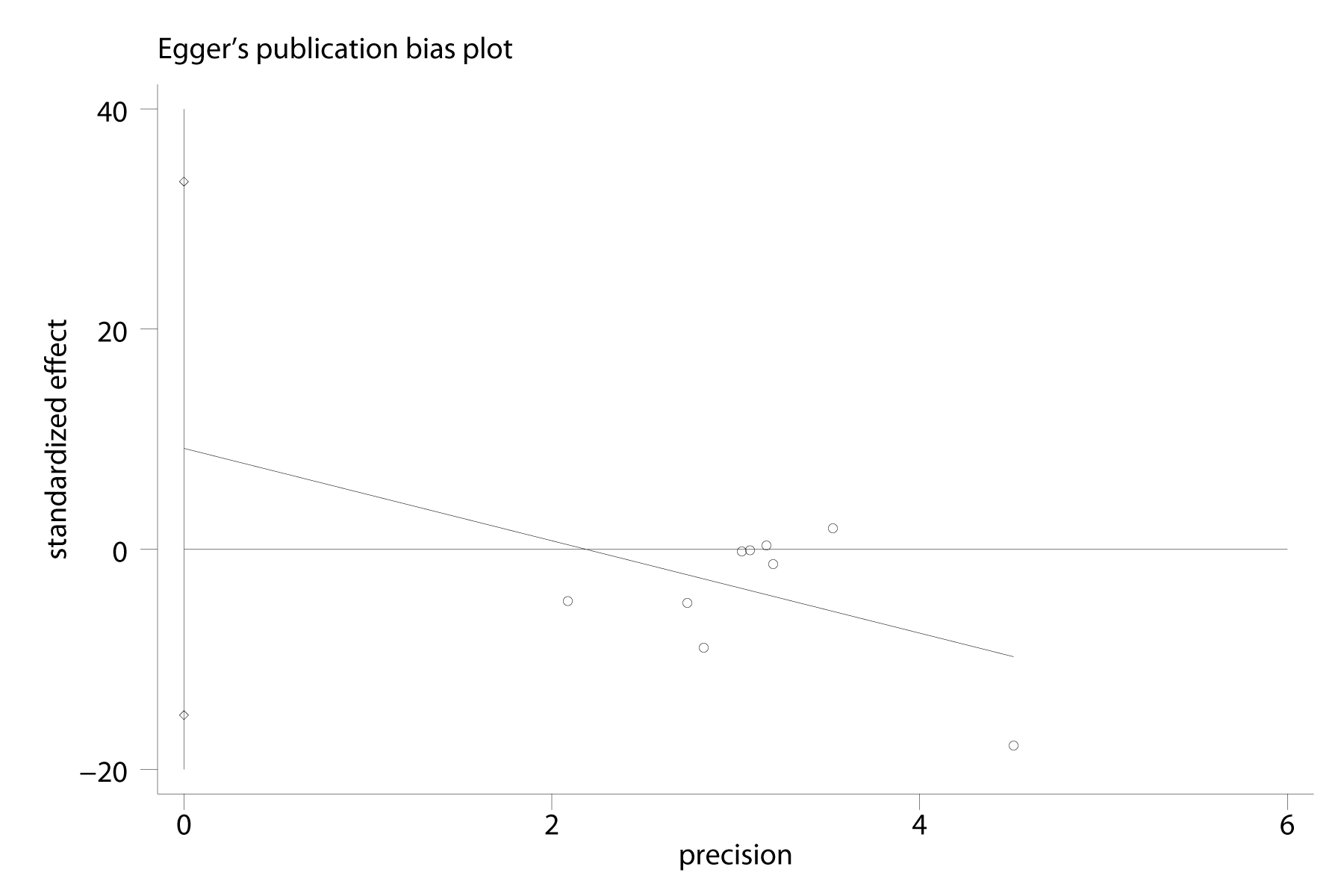

Supplement: S4 Fig — (TIF) [file pone.0132274.s004.tif]

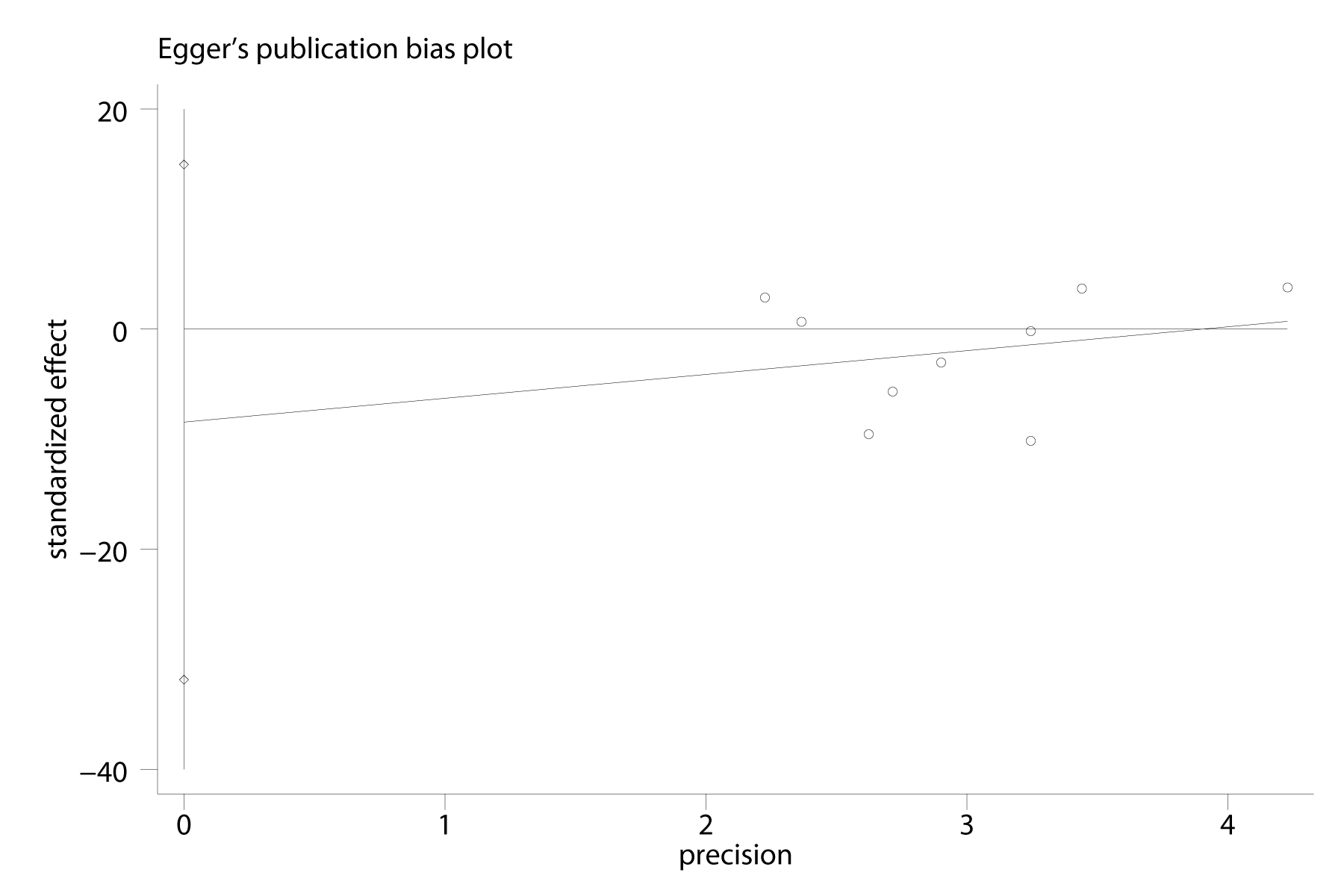

Supplement: S5 Fig — (TIF) [file pone.0132274.s005.tif]
