# Supplementary material for: The C825T Polymorphism of the G-Protein β3 Gene as a Risk Factor for Depression: A Meta-Analysis
Source: PLoS One. 2015 Jul 6;10(7):e0132274. doi: 10.1371/journal.pone.0132274 (PMC4493085; doi:10.1371/journal.pone.0132274)
Supplement: S1 File — (DOC) [file pone.0132274.s006.doc]

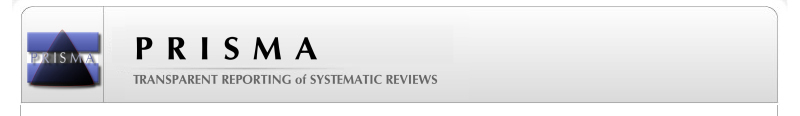
**PRISMA 2009 Flow Diagram**

**Screening**

**Included**

**Eligibility**

**Identification**

Records identified through database searching
(n = )

Additional records identified through other sources
(n = )

Records after duplicates removed
(n = )

Records screened
(n = )

Records excluded
(n = )

Full-text articles assessed for eligibility
(n = )

Full-text articles excluded, with reasons
(n = )

Studies included in qualitative synthesis
(n = )

Studies included in quantitative synthesis (meta-analysis)
(n = )
