# Supplementary material for: The C825T Polymorphism of the G-Protein β3 Gene as a Risk Factor for Depression: A Meta-Analysis
Source: PLoS One. 2015 Jul 6;10(7):e0132274. doi: 10.1371/journal.pone.0132274 (PMC4493085; doi:10.1371/journal.pone.0132274)
Supplement: S1 Table — (DOCX) [file pone.0132274.s009.docx]

**Table S1. Sensitivity Analyses for C-Allele versus T-Allele**

| **Study Excluded** | **P-value** | **Pooled ORs** | **95% Confidence Interval (CI)** | |
| --- | --- | --- | --- | --- |
|  |  |  | **Lower 95% CI Limit** | **Upper 95% CI Limit** |
| None | 0.002 | 1.39 | 1.13 | 1.72 |
| Alessandro | 0.003 | 1.43 | 1.13 | 1.80 |
| Anttila | 0.002 | 1.44 | 1.15 | 1.81 |
| Cao | 0.005 | 1.30 | 1.08 | 1.57 |
| Chen | 0.007 | 1.38 | 1.09 | 1.73 |
| Kunugi | 0.001 | 1.45 | 1.16 | 1.81 |
| Lee | 0.008 | 1.38 | 1.09 | 1.76 |
| Lin | 0.0003 | 1.47 | 1.19 | 1.80 |
| Peter | 0.009 | 1.35 | 1.08 | 1.69 |
| Xiao | 0.01 | 1.35 | 1.07 | 1.70 |
