# Supplementary material for: The C825T Polymorphism of the G-Protein β3 Gene as a Risk Factor for Depression: A Meta-Analysis
Source: PLoS One. 2015 Jul 6;10(7):e0132274. doi: 10.1371/journal.pone.0132274 (PMC4493085; doi:10.1371/journal.pone.0132274)
Supplement: S2 Table — (DOCX) [file pone.0132274.s010.docx]

**Table S2. Sensitivity Analyses for CC vs. CT+TT**

| **Study Excluded** | **P-value** | **Pooled ORs** | **95% Confidence Interval (CI)** | |
| --- | --- | --- | --- | --- |
|  |  |  | **Lower 95% CI Limit** | **Upper 95% CI Limit** |
| None | 0.003 | 1.53 | 1.15 | 2.04 |
| Alessandro | 0.009 | 1.55 | 1.12 | 2.16 |
| Anttila | 0.002 | 1.62 | 1.19 | 2.22 |
| Cao | 0.009 | 1.39 | 1.38 | 1.78 |
| Chen | 0.01 | 1.50 | 1.10 | 2.03 |
| Kunugi | 0.003 | 1.60 | 1.18 | 2.18 |
| Lee | 0.01 | 1.47 | 1.08 | 2.00 |
| Lin | 0.0005 | 1.64 | 1.24 | 2.17 |
| Peter | 0.01 | 1.47 | 1.08 | 2.00 |
| Xiao | 0.005 | 1.58 | 1.14 | 2.17 |
