# Supplementary material for: The C825T Polymorphism of the G-Protein β3 Gene as a Risk Factor for Depression: A Meta-Analysis
Source: PLoS One. 2015 Jul 6;10(7):e0132274. doi: 10.1371/journal.pone.0132274 (PMC4493085; doi:10.1371/journal.pone.0132274)
Supplement: S3 Table — (DOCX) [file pone.0132274.s011.docx]

**Table S3. Sensitivity Analyses for CC vs. TT**

| **Study Excluded** | **P-value** | **Pooled ORs** | **95% Confidence Interval (CI)** | |
| --- | --- | --- | --- | --- |
|  |  |  | **Lower 95% CI Limit** | **Upper 95% CI Limit** |
| None | 0.005 | 1.84 | 1.20 | 2.83 |
| Alessandro | 0.003 | 1.98 | 1.27 | 3.11 |
| Anttila | 0.005 | 1.96 | 1.23 | 3.11 |
| Cao | 0.02 | 1.61 | 1.10 | 2.36 |
| Chen | 0.02 | 1.79 | 1.12 | 2.88 |
| Kunugi | 0.003 | 1.99 | 1.26 | 3.13 |
| Lee | 0.02 | 1.80 | 1.11 | 2.92 |
| Lin | 0.001 | 2.03 | 1.33 | 3.12 |
| Peter | 0.02 | 1.69 | 1.09 | 2.63 |
| Xiao | 0.02 | 1.77 | 1.09 | 2.87 |
