# Supplementary material for: The C825T Polymorphism of the G-Protein β3 Gene as a Risk Factor for Depression: A Meta-Analysis
Source: PLoS One. 2015 Jul 6;10(7):e0132274. doi: 10.1371/journal.pone.0132274 (PMC4493085; doi:10.1371/journal.pone.0132274)
Supplement: S4 Table — (DOCX) [file pone.0132274.s012.docx]

**Table S4. Sensitivity Analyses for CC vs. CT**

| **Study Excluded** | **P-value** | **Pooled ORs** | **95% Confidence Interval (CI)** | |
| --- | --- | --- | --- | --- |
|  |  |  | **Lower 95% CI Limit** | **Upper 95% CI Limit** |
| None | 0.007 | 1.32 | 1.08 | 1.62 |
| Alessandro | 0.02 | 1.29 | 1.04 | 1.60 |
| Anttila | 0.005 | 1.39 | 1.10 | 1.75 |
| Cao | 0.05 | 1.23 | 1.00 | 1.53 |
| Chen | 0.01 | 1.30 | 1.05 | 1.60 |
| Kunugi | 0.006 | 1.35 | 1.09 | 1.66 |
| Lee | 0.04 | 1.25 | 1.01 | 1.55 |
| Lin | 0.003 | 1.38 | 1.12 | 1.71 |
| Peter | 0.01 | 1.32 | 1.06 | 1.64 |
| Xiao | 0.74 | 1.41 | 1.14 | 1.75 |
