# Supplementary material for: The C825T Polymorphism of the G-Protein β3 Gene as a Risk Factor for Depression: A Meta-Analysis
Source: PLoS One. 2015 Jul 6;10(7):e0132274. doi: 10.1371/journal.pone.0132274 (PMC4493085; doi:10.1371/journal.pone.0132274)
Supplement: S5 Table — (DOCX) [file pone.0132274.s013.docx]

**Table S5. Sensitivity Analyses for CC+CT vs. TT**

| **Study Excluded** | **P-value** | **Pooled ORs** | **95% Confidence Interval (CI)** | |
| --- | --- | --- | --- | --- |
|  |  |  | **Lower 95% CI Limit** | **Upper 95% CI Limit** |
| None | 0.02 | 1.54 | 1.08 | 2.18 |
| Alessandro | 0.006 | 1.65 | 1.15 | 2.34 |
| Anttila | 0.02 | 1.59 | 1.09 | 2.33 |
| Cao | 0.04 | 1.41 | 1.01 | 2.04 |
| Chen | 0.03 | 1.54 | 1.04 | 2.28 |
| Kunugi | 0.009 | 1.63 | 1.13 | 2.36 |
| Lee | 0.03 | 1.57 | 1.06 | 2.33 |
| Lin | 0.01 | 1.62 | 1.12 | 2.35 |
| Peter | 0.05 | 1.42 | 1.00 | 2.03 |
| Xiao | 0.05 | 1.40 | 1.00 | 1.99 |
